# Using a Mobile Messenger Service as a Digital Diary to Capture Patients’ Experiences Along Their Interorganizational Treatment Path in Gynecologic Oncology: Lessons Learned

**DOI:** 10.2196/52985

**Published:** 2024-07-29

**Authors:** Eleonore Baum, Christian Thiel, Andrea Kobleder, Daniela Bernhardsgrütter, Ramona Engst, Carola Maurer, Antje Koller

**Affiliations:** 1 Institute of Applied Nursing Science School of Health Eastern Switzerland University of Applied Sciences St.Gallen Switzerland; 2 Department of Epidemiology and Public Health Swiss Tropical and Public Health Institute Allschwil Switzerland; 3 Faculty of Medicine University of Basel Basel Switzerland; 4 Institute for Information and Process Management Eastern Switzerland University of Applied Sciences St.Gallen Switzerland

**Keywords:** mobile apps, computer security, confidentiality, data collection, oncology, breast neoplasms, mobile phone

## Abstract

A digital diary in the form of a mobile messenger service offers a novel method for data collection in cancer research. Little is known about the things to consider when using this data collection method in clinical research for patients with cancer. In this Viewpoint paper, we discuss the lessons we learned from using a qualitative digital diary method via a mobile messenger service for data collection in oncology care. The lessons learned focus on three main topics: (1) data quality, (2) practical aspects, and (3) data protection. We hope to provide useful information to other researchers who consider this method for their research with patients. First, in this paper, we argue that the interactive nature of a digital diary via a messenger service is very well suited for the phenomenological approach and produces high-quality data. Second, we discuss practical issues of data collection with a mobile messenger service, including participant and researcher interaction. Third, we highlight corresponding aspects around technicalities, particularly those regarding data security. Our views on data privacy and information security are summarized in a comprehensive checklist to inform fellow researchers on the selection of a suitable messenger service for different scenarios. In our opinion, a digital diary via a mobile messenger service can provide high-quality data almost in real time and from participants’ daily lives. However, some considerations must be made to ensure that patient data are sufficiently protected. The lessons we learned can guide future qualitative research using this relatively novel method for data collection in cancer research.

## Current Perspectives on Digital Diaries in Cancer Research

### Overview

Asking research participants to write their experiences down in a diary generates different data from those that might be obtained from qualitative interviews. Using ambulatory assessment (AA) methods in the form of diary writing allows for the collection of rich data during the patient’s daily life almost in real time [1,2]. AA is a research tool that has grown dramatically in popularity, particularly in the last decade [2,3]. AA refers to an array of assessment approaches that include the experience sampling method (such as paper-pencil diaries) or ecological momentary assessment (EMA; such as digital diaries on mobile phones) [3,4]. AA ensures that data collection occurs within the patient’s individual natural environment and context. This improves data validity as opposed to more traditional assessment methods that often take place in artificial environments [2,5]. Moreover, data collection methods such as the experience sampling method or EMA have the potential to reduce recall bias [6,7].

AA has commonly been used to collect structured quantitative data, for instance, using software to support clinical research projects, such as REDCap (Research Electronic Data Capture; Vanderbilt University) [8]. In cancer research, AA techniques have been applied using specifically developed mobile applications functioning as diaries, for instance, to track clinical symptoms [9] or to assess self-report physical activity [10,11]. In addition, AA or EMA in oncology research holds promise to improve the understanding of patients’ symptoms and quality of life by taking their natural environment into consideration [12].

However, such techniques can also be a useful data collection tool for qualitative research [4]. Applying AA in mental health research is well established. However, less is known about its feasibility and added value for oncology research [12].

Diaries in digital format can offer a variety of data, such as videos, audios, and photos, which can be recorded [13]. More specifically, digital diaries in the form of a mobile messenger service or application enable remote data collection with the possibility of real-time feedback on participant activity, thereby improving collaboration between members of the research team and participants. Telling a patient’s story with the help of mobile digital devices in the research context encourages a detailed description [14] and holds promise as a participatory research practice [15,16]. Capturing patients’ stories is especially useful in qualitative research to explore participants’ lived experiences with health care [17]. Moreover, the treatment of gynecological and breast cancer is multiprofessional and interorganizational, as it mostly includes a combination of surgery, chemotherapy, radiation, and targeted therapies. Patients have numerous appointments, often in several different locations, with their experiences potentially getting lost in retrospective interviews.

### Background on the Original Study

This viewpoint paper was inspired by the experiences we acquired in an original study with the aim of examining the meaning of trust, interprofessional collaboration, and the role of the advanced practice nurse in gynecological oncology in the treatment path of women with gynecological cancer. Detailed study procedures and results will be provided elsewhere and are not the focus of this viewpoint. We conducted a mixed methods study [18] and chose for the qualitative part an interpretive phenomenological approach in accordance with van Manen [19]. A total of 12 women (aged 27 to 61 years) were recruited in 2 oncology clinics by 2 advanced practice nurses. Most participants had a higher qualification (n=9, 75%) and were in the cancer stage IA (n=4, 33%), followed by stage IIA (n=3, 25%), according to the Union for International Cancer Control.

We asked participants to share their experiences using a mobile messenger service installed on a tablet as a digital diary from diagnosis to follow-up. The mean study duration among the 12 participants was 10.5 (SD 4.1, range 2-16) months.

The diary was unstructured to reflect participants’ individual experiences and perceptions around the phenomena of trust and interdisciplinarity. This meant the participants did not receive specific questions from the research team but sent us their perceptions on trust and interdisciplinarity at varying time intervals and with varying subjective content depending on their circumstances and differing treatment paths. The participants were informed that they should view and use Threema (Threema GmbH) similarly to an actual paper-based diary, writing down their inner reflections and perceptions. This also meant that participants did not necessarily expect any response to their diary entries or prompts from the research team on a regular basis. However, we found that this flexible approach met recommendations for the successful integration of EMA techniques, such as permitting continuous researcher and patient interaction, sending questions, reacting to responses, and debriefing after the completion of treatment phases [20].

Along with the digital diary, we conducted repeated semistructured interviews, and participants filled in a structured questionnaire at each treatment phase (ie, diagnosis, surgery, chemotherapy, radiation, and follow-up). Through this triangulation of methods, we were able to generate richer data [21].

### Ethical Considerations

The study was approved by the responsible ethics committee of northwest and central Switzerland (registration number 2021-00730). All patients provided written informed consent before enrollment in the study. Participants were informed that participation in the study was completely voluntary and that nonparticipation or withdrawal was possible at any time once participation had begun without any consequences concerning further care and treatment or employment. Texts sent via the messenger service were encrypted before data analysis to ensure confidentiality. The encryption key was kept in the Institute of Applied Nursing Science, separate from other data. Participant data were stored on a password-protected drive. The patients received no compensation for participation. However, each patient received a tablet to be used as a digital diary. The tablet was given to the patients as a gift. With regard to our digital diary approach, the ethics committee deemed it important for us to report in detail how the data were encrypted and stored to ensure data protection.

### Rationale for Using Digital Diaries in Cancer Research

For the original study, we chose the digital diary method via a mobile messenger service mainly for 3 reasons [22]. First, cancer treatment may impair physical and cognitive function. Therefore, we tried to keep patient burden as low as possible and minimize the time between women’s experiences and their reporting. Accurate reporting is particularly important in view of the complex treatment that involves numerous appointments with several health care professionals [23]. By solely relying on interviews conducted retrospectively and in light of any potential impairment of cognitive function due to the treatment, the accuracy of the women’s reports may else have been compromised. Second, trust is highly subjective by nature. It takes time to develop, may change with time, and is influenced by previous collaboration experiences [24]. To reflect the participants’ lived experience and the phenomenon of trust over time, the diary method using mobile messenger services, such as the ones many people already use on their mobile phones, appeared particularly appropriate [25]. It seemed to fit the phenomenological methodology, as mobile messenger services are already part of our day-to-day experience, as opposed to additional diary applications or software. Using specific digital diary software with structured prompts could be viewed as an added hurdle to be part of participants’ daily lives. Therefore, messenger services show promise in capturing nuances of patients’ lived experiences along the illness and treatment journey [26].

The digital diary method also allows participants’ testimonies of their lives to shape the direction of the research, thereby addressing power imbalance [27]. Third, the technical solution provided advantages such as responsive and transparent data collection and password protection, which would prevent reading by others and contribute to data security. However, research has also found pitfalls when using mobile messenger services in the context of health care in the past. A recent scoping review on the use of the WhatsApp (Meta Platforms Inc) messenger found research ethics were not considered adequately. The authors deemed this to be concerning, given the controversies WhatsApp has faced regarding data protection in detail end-to-end encryption [28]. In addition, for health researchers, technical solutions such as end-to-end encryption might not be obvious, and the need for technical support might be underestimated. Data security policies and regulations are constantly adapted in Switzerland and other countries in Europe. Digital modes of data collection add new aspects to consider when protecting the sensitive data of study participants. From a practical point of view, the interface of digital applications on electronic mobile devices requires other practical skills and preparations of the researchers than classical data collection methods. For example, IT support is needed for planning and maintaining the running systems and for troubleshooting when problems occur.

In summary, using a mobile messenger service for qualitative data collection seems an opportune way to collect data nowadays. However, it poses some questions, particularly with regard to data quality, practical aspects, and data protection, that need to be considered when choosing this option for clinical studies.

### Aim

The purpose of this viewpoint paper is to discuss the lessons we learned from applying a digital diary as a data collection method by sharing our experiences, some solutions, and questions that we encountered while using this method. We aimed to provide useful information to other researchers who consider digital diary methods for their research, especially in the context of oncology care.

## Methods

The paper covers our experiences on three main topics: (1) data quality, (2) practical aspects, and (3) data protection. Our initial considerations around these 3 topics and how we approached them are described in the following sections.

### Data Quality

In terms of data quality, we paid particular attention to the extent to which the data collected with the mobile messenger service conformed to the paradigm of phenomenological methodology. In doing so, we held continuous team meetings, recorded the discussions, and analyzed them for this paper. As a more universal approach to data quality, we used the quality criteria of Lincoln and Guba [21], namely credibility, transferability, dependability, and confirmability. In addition, we considered the criteria of authenticity [29]: fairness, ontological authenticity, educative authenticity, catalytic authenticity, and tactical authenticity. How we incorporated these criteria into our research method is elaborated in the Key Lessons Learned section.

### Practical Aspects

Practical aspects of planning, setting up, and maintaining the mobile devices as well as the software were continuously recorded by the research team in a field protocol. Practical solutions and questions that were addressed are reported in this manuscript. The analysis of the protocols and data security was carried out in joint discussions with the primary team (experienced researchers with a clinical nursing background), including an IT expert.

### Data Protection

Because of the interactive nature of the messenger service, we had to consider the privacy protection of both parties, the patients and the research staff. Essential requirements for messenger services were derived from the European Union (EU) data protection legislation. The introduction of the EU General Data Protection Regulation (GDPR) has an impact on everyone who moves on the web. Although this is an EU regulation, it nevertheless has an extraterritorial effect, as companies from nonmember states are also obliged to implement it under certain circumstances. Switzerland’s revised data protection act entered into force on September 1, 2023, and approximates the GDPR.

## Key Lessons Learned

### Overview

We compared certain evaluation criteria for each messenger to then selected a suitable messenger service. The three main areas of focus namely (1) data quality, (2) practical aspects, and (3) data protection (including data privacy and information security), were interrelated, and decisions in one area most often needed some considerations for one of the others. For instance, it was considered practical by some research team members to text patients from their personal mobile devices, but this led to new data protection issues.

### Data Quality

We ensured *credibility* by interacting with our participants on a regular basis through the messenger service over a lengthy period, from the phase of cancer diagnosis to follow-up several months later. The interactive nature of the messenger service allowed us to enrich the content the participants posted nearly in real time, thereby largely circumventing any recall bias. However, we found that this bias could not be completely omitted. During data collection, several participants forgot to send us their messages, especially in the last phase of data collection during follow-up, or were simply too burdened by the side effects of the treatment to update us regularly. However, we felt these missing data could be mitigated through data triangulation. For instance, when we conducted the semistructured interviews at each treatment phase, we could inquire about information that had potentially gone missing in the diary. Moreover, we paid particular attention to the aspect of *social desirability* In our experience, we found this possible bias to be rather negligible, as all participants felt comfortable sharing positive and negative information around their experiences. The interaction with the patients on the mobile messenger service allowed us to inquire what was meant if a certain message was ambiguous. We entered probing questions into the messenger service to delve deeper when certain information that touched upon our research question was provided. In addition, during the interviews, we gave respondents the opportunity to correct misinterpretations we might have made of their digital diary messages, thereby triangulating and cross-checking the data. We ensured *transferability* by encouraging participants to share their narrative with us. However, sometimes, individual requirements called for the application of pragmatic decisions. For instance, we allowed one of the participants to send us photos of handwritten messages, as she mentioned feeling less comfortable typing a lot of information into the tablet. However, she used this option only twice before switching to email. We considered *dependability* and *confirmability* by, for example, noting down common responses of the research team in a separate file. (For example, “Dear participant, thank you for your detailed descriptions. We will consider them carefully.”) By archiving our responses and discussing them among ourselves, we ensured that our reactions were similar across patients in case of comparable situations. Exchanges with study participants outside of the messenger service (including quick telephone calls for technical reasons or setting an interview date) were recorded. To limit researcher bias, we avoided steering the participant’s narrative too strongly to capture the participant’s lived experience. This was particularly relevant for probing questions that we entered into the messenger service. In this regard, we found it particularly helpful that each participant chat group was supervised by 3 researchers and that responses could, therefore, be discussed.

Moving on to the 5 dimensions of authenticity according to Lincoln and Guba [29,30], we aimed at achieving *fairness* through our prolonged engagement with participants and regular peer debriefings with fellow researchers who were in the chat groups. We established *ontological and educative authenticity* by allowing ourselves as researchers to be “educated” by our participants, taking on an emic perspective. The ongoing dialogue with participants helped achieve a sense of familiarity in the researcher-participant relationship and led to an effective hermeneutic circle. Regarding *catalytic authenticity,* we discussed and defined how we interacted with study participants throughout the research process, especially how we communicated over the messenger. When situations that we had not fully anticipated beforehand arose, for instance, certain technical difficulties of participants, we found solutions through discussion and joint decision-making in the research team. To ensure *tactical authenticity,* we spoke to participants over their personal telephone before every initial interview, inquiring whether they felt empowered to fully communicate and share their experiences with us over the mobile messenger service. Participants could also exercise control on how frequently they interacted with us, when they sent us messages, and what content they decided to share with us.

Overall, we found that the use of a mobile messenger service for the digital diaries fully met our expectations in view of data quality. The richness and authenticity of data that were generated via this method were exceptionally high and ensured the credibility of the data. We felt that this method yielded a feasible and personal way to accompany a woman directly after her gynecological or breast cancer diagnosis and get to know her lived experience during her treatment almost in real time. In our study, the digital diary data complemented the semistructured interviews most favorably. We found that the contact via the mobile messenger service moved the starting point in each interview much closer to the focus of our primary research question on trust and interdisciplinarity, as the researcher already knew eminent details of the participants’ lived experience. This was especially relevant in the initial study phases, when participants experienced more uncertainty and emotions.

### Practical Aspects

Field protocols mainly yielded 2 practical interrelated groups of things to consider when using digital diary data collection: technical aspects and the interactive nature of the messenger service.

### Technical Aspects

In our research context, it was important that the selected messenger service was flexible, was popular in terms of its recognition value, and had the greatest possible compatibility with common operating systems, such as Android (Google LLC) or iOS (Apple Inc). The messenger service needed to be compatible with not only the tablets provided but also the user’s smartphone. Data security played a major role when choosing a messenger service, and the most important criteria are described later in this paper in the Data Protection section. Costs also guided our decision because tailored mobile diary apps can be expensive compared to more affordable messenger services. Finally, we selected the Swiss mobile messenger service Threema.

As tablets were gifted to the participants during recruitment, all participants chose to use the tablet instead of their mobile phone. One of the participants mentioned that she wanted to keep the “disease and her private life separate.” Another reason for patients not choosing their own mobile device for posting messages might have been that the research assistants in both settings did not emphasize this possibility sufficiently. The tablets were provided fully set up and preinstalled. However, the tablets were not as much part of patients’ everyday life experience as we had hoped. Notably, at the end of data collection, some participants mentioned not wanting to continue to use the gifted tablet for other purposes because they associated it with their disease. Indeed, one of the participants mentioned having given the tablet to a relative. None of the tablets were returned to the research team.

During data collection, patients sometimes forgot to bring the tablet with them to certain appointments (eg, radiation appointments) to document their experiences. Had they been using their smartphones to communicate with us, we assume this would have happened less frequently. In some cases, the research team had difficulties reaching participants (1) when the first contact was problematic (refer to the subsequent discussion about Wi-Fi connection) or (2) when the participants had not recorded their experiences for some time. According to the study protocol, contact should be held solely via the mobile messenger service. However, as some participants did not boot their tablet regularly, this way of communication was not always reliable. Instead, we established an initiating phone call as well as email contact in individual cases for first and ongoing contact. If messages were not sent to the research team via the messenger service for >3 weeks, the research team tried to contact the participant via the messenger service and then via the contact person in the respective setting, and if this did not suffice, the research team tried to establish contact via phone or email. However, we discussed how often or in which intervals phone calls might be appropriate if patients did not send us a message for a while.

This way, we were able to keep in contact with all participants and, at the same time, respect their personal life as much as possible. No participant dropped out of the study, despite the longitudinal character with a mean study duration of 10.5 months and no financial incentive. All in all, this confirms a high level of feasibility of the data collection method from the participants’ side.

Some decisions entailed situations that were not anticipated by the research team. For example, as the chosen mobile messenger service was distributed via a common mobile app store, a new account for each tablet needed to be created by the team, which affected complex password management, among other things. The payment method needed to be compatible with the standards of the project’s financial control. In addition, the used mobile app store account and payment method had to be safe from fraud and unlawful use by subsequent app users. As a solution, mobile app store vouchers were used instead of entering the details of a credit card.

The selected messenger service stores data on each mobile device rather than in the cloud. In one instance, a message was not received by one of the research staff due to synchronization problems in the messenger service. As we had regular team discussions and chats were hosted by 3 members of the research team, this problem was soon discovered, and the message could be retrieved. Another time, we encountered a synchronization problem related to the decentralized data backup on participants’ mobile devices: some messages were received with the incorrect date and time stamp. Research team members in the chat quickly realized this issue when messages were sent in the wrong sequence. Having several researchers present in each chat was a definitive advantage. Once we realized the discrepancy, the administrator of the chats synchronized chat groups regularly, and we no longer encountered this issue.

Although generally not a problem, establishing a Wi-Fi connection on the tablet did require some basic technical knowledge. In one situation, initial Threema messages after recruitment were not recorded via the messenger service on the tablet until the participant had her husband connect the tablet to her home Wi-Fi. Some data from the early treatment phases were lost due to this technical issue. The research team could not offer remote support for this kind of problem, as each Wi-Fi router might be different. However, as connecting mobile devices to home Wi-Fi was a known task for most participants, this was not problematic and could be solved without much burden for the participant.

Another practical technical issue that was not fully anticipated by the team was the practicability of producing and sending text messages on a tablet. For example, participants frequently mentioned that the autocorrection feature wrote “odd” words, and they were not always capable of disabling the function. As a result, they needed to recheck each message and correct mistakes manually, which increased time and effort for data generation and caused frustration for one of the participants. We would recommend disabling the autocorrection mode before handing out the device to participants or providing a guide regarding how to do this manually. In addition, the handling can differ between mobile devices. For example, a participant suggested changing to writing emails from her private laptop instead of using the messenger service on the tablet because she could not become used to the tablet swipe function compared to the keyboard on her laptop. We decided that she can send her emails to a protected server of a research team member.

Surprisingly, the time-saving method of sending voice notes via Threema was hardly ever used, even though research assistants suggested this method in the supporting phone calls. Therefore, we assume that this method of messaging posed a greater barrier to participants than text messaging. As a result, data generated by Threema consisted mostly of written text messages. One of the participants elaborated on her dislike of sending voice notes. She mentioned that it was important for her to reread the text she had written before sending it to us.

### The Interactive Nature of the Messenger Service

The interactive nature of the messenger service triggered our hypothesis that participants might expect some feedback on their posts. In the study protocol, the team planned to ensure so-called impartial feedback to balance the abovementioned “steering” of the data with the amount of data that were derived. We ensured timely feedback by building teams of 3 researchers for each chat. One person per chat was in charge of answering in an impartial manner. In this context, impartial meant that the responses did not reinforce emotional topics and used the participant’s own words whenever possible. A common answer to a post was, for example, “Thank you for this interesting message. I am curious how xyz will turn out.” During data collection, we repeatedly discussed how our answers may influence the participants’ posts. We also considered whether certain replies may be used to direct posts intentionally to generate more data that focused on the research question. After consulting an external expert for phenomenology, we decided on a mode of intentionally “steering” participants’ posts to generate richer data in view of the research question.

For the Swiss context, we needed to choose the teams according to their language-speaking abilities, as the study sites were from 2 different language regions (Swiss German and French). As the chats were backed up regularly on local servers, a person in charge was chosen to oversee this task. This team member had to be extra careful not to miss any messages; otherwise, some messages might not have been included in the transcript (refer to the Technical Aspects section). In addition, participants had to be informed regarding who was able to read their posts and why 3 team members were part of the chat group. As some participants inquired whether the other participants were able to read their posts in the messenger, we realized that confidentiality needed to be explained in more detail. As is standard in qualitative clinical studies, we informed patients that the research team was not giving any medical advice. Some discussions were necessary to balance the neutrality of the answers and responsiveness when some participants revealed that they had acute health problems. We stored standard answers with emergency numbers of the treating departments for each study site, for which we coordinated with the on-site contact persons.

Another aspect of the personal attendance of the chat groups was discussed frequently in the team: some patients posted mainly in the evening and late at night. Some research team members used distinct ringtones; others muted the messenger service to avoid receiving potentially upsetting messages in their spare time. This was possible, as some researchers did not use Threema in their personal life at the time of the study. However, if message notifications were not muted, posts reached us outside of working hours. Balancing the pros and cons, most of us stuck to our choice to use our personal mobile phone for chat supervision. This seemed more convenient and allowed the team members to report in a timely manner, becoming somewhat part of patients’ lived experience.

### Data Protection

In view of data protection, we found that several aspects of data privacy and information security had to be considered for the selection of the mobile messenger service [31-33]. The criteria in Textbox 1 indicate when a messenger service may be suitable for exchanging sensitive health-related data on a more general level.

**General criteria when choosing a messenger service for data collection.**

**General criteria**

- Privacy by design—data security and metadata sparing are already considered in the development phase of the messenger. Where there are no data, there can be no misuse of it.
- Open source—the app’s source code is publicly available and can be viewed by third parties.
- End-to-end encryption—only the chat participants can view the exchanged information. Even the service operator has no way of decrypting it.
- Self-hosting—the messenger is run on the institution’s own server (on premises) to meet its own security requirements and retain sole data sovereignty. In the context of temporary research projects at universities, self-hosting and administrability are not always possible. In that case, at least the operation of the solution (server) should be subject to the respective country-specific data protection legislation.
- Data protection conformity—the messenger’s functionality fully complies with the strict regulations of the General Data Protection Regulation, which means that the misuse of confidential health care data can be ruled out.
- User-friendliness and intuitive handling—if the requirements of user-friendliness and functional scope are not met, even the most secure messenger service is worthless and may promote the emergence of applications and services without explicit IT department approval.

These generic requirements lead to a comprehensive checklist that can be used to evaluate current and future messengers in terms of their suitability for handling sensitive health data. The checklist and the definitions of the different criteria are provided in the Multimedia Appendix 1. The Messenger Evaluation Checklist is applied to 5 publicly available messenger services in the Multimedia Appendix 2, allowing for comparisons of different technical aspects.

According to this evaluation, various messengers could have been used for the application scenario of our study (or similar studies handling sensitive data). It is also to be expected that more suitable messengers will be developed or that existing providers will close their weak points regarding data protection and the security of customer data. In the context of this study, which was conducted in Switzerland, we selected the Swiss app Threema from the suitable messengers for three main reasons:

1. Threema was the first major messenger service to commit to data protection and privacy.
2. Threema does not require personal data (data sparseness).
3. The messenger offers a number of similar functions to those offered by WhatsApp, such as sending voice notes or photos, which also catered to different communication preferences of patients.

First, as an important mechanism for data protection, Threema uses secure “end-to-end” encryption. This is comparable to specific AA applications, such as movisens. This was relevant to our study because we did not provide a SIM card, and we found that transmission via Wi-Fi would suffice. Participants were able to type their posts in the messenger service offline, which were sent as soon as the tablet was connected to Wi-Fi. We left it to the participants to decide which Wi-Fi they used. However, we could not ensure data security for open-access Wi-Fi, such as hospital Wi-Fi or restaurant Wi-Fi. One solution might have been a virtual private network connection. However, as we had to balance technical skills and usability with data protection, the end-to-end encryption of the messenger service was considered safe enough.

Second, in terms of data sparseness, Threema does not ask for any data when registering and adheres to strict data protection guidelines. Moreover, as the application was preinstalled on the tablets by the research team, participants did not need to register using their private phone number. The address book is not by default synchronized and matched with existing messenger users. If data are collected, they are deleted again immediately. Text messages and media are stored only on the end devices unless the user chooses the backup function. The servers then only have the function of a relay station: messages and data are forwarded but not permanently stored. As Threema cannot view or process messages, users retain control over their messages.

Third, fortunately, Threema not only meets the functional requirements in our study but also adequately protects the personal data of all chat group participants (study participants and research staff). Moreover, according to the terms of service, Threema does not store any metadata that could be used to identify the message content. Threema uses data only to provide the necessary service, emphasizes data privacy, and minimizes data collection. There is no evidence that Threema uses data for modeling or other purposes.

In addition to the 3 reasons that led to its selection, Threema fulfills many other requirements, as listed in the Multimedia Appendix 1 and evaluated in the Multimedia Appendix 2.

## Discussion

### Principal Findings

In this viewpoint paper, we intended to report the lessons we learned using a mobile messenger service to generate data for digital diaries in an oncology setting. Overall, we found that using a mobile messenger service such as Threema as a digital diary was feasible and highly valuable for data collection, and we never regretted our decision. The flexibility around diary content and timing of messages was a major strength of the study, particularly for this patient population facing significant challenges. Although we did not send regular prompts, the diary generated rich data. The challenges that we met during our study showed us that technical support needs to be carefully considered while planning and conducting such research. Technical aspects should entail software and hardware in view of the suitability of the backend and the usability of the front end. These considerations might include not only the choice of the software and means of data protection but also practical front-end considerations, such as technical support if a device is not working or the practicality of writing long, emotional messages with a touchscreen keyboard.

### Data Quality

In our study, it was particularly important for us to collect high-quality data that ensured we could take part in participants’ lived experiences. Using a mobile messenger service provided us with rich descriptions that participants did not necessarily share with us during the interviews. Researchers in another study came to similar conclusions, where they introduced a chatbot for patients with breast cancer. They found that patients revealed more intimate information and shared thoughts about sexuality and hair loss [34]. In another study, the authors confirmed that participants felt more at ease sharing private information over the messenger because of anonymity and privacy considerations. The authors concluded that messenger services also have the potential to facilitate trust and, therefore, the collection of more in-depth data, especially in longitudinal studies [35], which we also observed in our study. We found in our study that interacting regularly with participants over the messenger service made the exchange more personal. We felt as though we were taking part firsthand in the participants’ experiences almost in real time. On the one hand, participants at times forgot to update us via the tablet, and some participants expressed a wish to keep their private life and the disease separate, particularly in the follow-up phase. However, we still felt that this method achieved a level of nearness we could not have achieved through interviews or questionnaires only. In addition, we felt that the mobile messenger service aided us in circumventing the issue of recall bias, which can be an issue for other methods of data collection [6]. This is especially important for cancer research, considering chemotherapy-related cognitive impairment, such as memory deficits [36,37].

Furthermore, and in line with findings from the study by Herron et al [38], the digital diary entries gave us clues as to how the interviews could further deepen the participants’ accounts of their experiences. In addition, we informed the participants which member of the team was primarily responsible for interacting with them within the messenger. This team member usually also conducted all interviews, which additionally fostered trust between the researcher and participant.

### Practical Aspects

In our study, we reflected on technical aspects as well as researcher and participant interaction. Participants were eager to send us Threema messages at the start of the study, but at certain points in their treatment path, especially during radiation and follow-up, engagement was an issue. Other research suggests that a high attrition rate and continuous engagement are key challenges in health care studies that rely on mobile applications [39]. However, researchers found that personal contact, such as receiving response messages from researchers, made participants feel valued, and they were more likely to complete data collection [40]. It was paramount in our study to be able to interact with participants and to use other mediums when Threema communication declined or came to a halt. As we had very little missing data and no dropouts, this somewhat laborious method paid off in our study. We think that this may also be the reason why we had no dropouts. This is particularly worth mentioning, as the study took place over a long period during which several patients did not feel well. The low dropout rate coincides with the strengths highlighted by Trull and Ebner-Priemer [3] on self-report AA. The authors argued that patients with severe illness have demonstrated good compliance in AA studies and are willing to share honest reports of their experiences.

### Data Protection

Data protection was one of our main considerations when we selected the messenger service Threema. Weis et al [41] found in their qualitative study that an important concern for patients with cancer was data security as well as confidentiality. We made sure that study participants were well aware of all relevant data protection aspects. Interestingly, none of the patients with cancer expressed any unease or worry in this direction when communicating with us over the messenger service.

One concern for the researchers of this study, however, was the data security for open-access Wi-Fi, as we did not provide patients with a SIM card. Cyber security and issues of cyber security vulnerability among Android devices have also been reflected upon in other health care research [32,42]. Mierzwa et al [43], for instance, recommend considering the cyber-risk likelihood and a consequence analysis when using a certain technology in health care. They found that WhatsApp is not an adequate tool to share clinical information due to its noncompliance with the GDPR and Health Insurance Portability and Accountability Act rules. In their opinion, health care organizations and physicians should abandon WhatsApp, moving toward secure messaging apps that are able to maintain the confidentiality and security of patient data. However, these works mostly considered tools for communication within health care organizations and for communication between their employees.

### Potential Pitfalls

Several different mobile messenger services could have been used in our study. We did not conduct an in-depth analysis of all available applications beforehand but instead selected Threema out of 3 main publicly known applications based on the 3 reasons mentioned in the Data Protection section.

We did not encounter any major pitfalls in the process of ethical clearance that diverged from our previous non–digital diary studies with patients with cancer. However, it is important to note that ethical clearance requirements may differ greatly, even within the same country. The mobile messenger service used in this study is based in Switzerland, the same country where the study was conducted. This may have influenced the process.

We cannot rule out the possibility that the technical nature of the digital diary might have resulted in a slightly biased sampling, with technically affine persons feeling more inclined to participate in this kind of study. However, we found that age and educational level showed a certain diversity.

The research team of this study did not have a professional background or expert IT knowledge. Study participants also did not receive any specific training to use the mobile application, as recommended by Daniëls et al [44]. We could only provide remote support via phone when the study participants experienced technical difficulties. In such cases, we referred participants to the recruiting nurse, who also lacked the technical expertise to help with participants’ mobile devices. Mostly, we could solve the problems. However, if not, we indeed jointly found individual pragmatic solutions, such as allowing the use of pictures of handwritten messages or using a keyboard and emailing diary messages. One might argue that the different modes of data collection as well as the unstructured setup of the diary in comparison to more conventional AA applications led to a slight imbalance in data quality. We checked whether a certain amount of “closeness” to the patient’s lived experience might have been lost due to this decision. In this particular case, for example, the switch to email was successful, as the participant immediately started sending us more detailed reports more frequently. It remains unclear whether the data collection method described here was superior or inferior to other data collection methods. However, we found that it led to a certain amount of “felt proximity” to participants’ lived experiences. As this was precisely the goal of our study, we found that using the digital diary method served our purpose quite adequately.

### Conclusions

We recommend using a mobile messenger service for digital diaries in studies because they can generate timely and rich data that represent patients’ lived experiences. This data collection method also shows promise for generating high-quality data over a longer period. Interacting and engaging with patients regularly over the messenger service may not only facilitate patients’ truthful responses but also greatly aid participant retention. We consider the digital diary method to be suitable for cancer research because it allows researchers to closely follow patients and partake in their experiences in each treatment phase in a timely manner. Last but not least, the combination of methods contributed to the 100% retention rate.

The choice of a high-quality messenger service is particularly important for researchers. We believe that our insights on data quality, practical aspects, and data protection provide details on how researchers may use this method to its best potential. We feel that more work may be needed to answer the questions that remained unsolved in our study.

## Appendix

Multimedia Appendix 1 Messenger evaluation checklist.

Multimedia Appendix 2 Exemplary analysis of 5 messengers.

## Abbreviations

AA: ambulatory assessment
EMA: ecological momentary assessment
EU: European Union
GDPR: General Data Protection Regulation
REDCap: Research Electronic Data Capture

## Data Availability

The relevant data are in the paper or in the Multimedia Appendices 1 and 2.

## References

[1] Schneider S, Stone AA (2016). Ambulatory and diary methods can facilitate the measurement of patient-reported outcomes. Qual Life Res.

[2] Trull TJ, Ebner-Priemer UW (2020). Ambulatory assessment in psychopathology research: a review of recommended reporting guidelines and current practices. J Abnorm Psychol.

[3] Trull TJ, Ebner-Priemer U (2013). Ambulatory assessment. Annu Rev Clin Psychol.

[4] Shiffman S, Stone AA, Hufford MR (2008). Ecological momentary assessment. Annu Rev Clin Psychol.

[5] Fahrenberg J (1996). Ambulatory assessment: issues and perspectives. Ambulatory Assessment: Computer-Assisted Psychological and Psychophysiological Methods in Monitoring and Field Studies.

[6] Stone AA, Shiffman S (2002). Capturing momentary, self-report data: a proposal for reporting guidelines. Ann Behav Med.

[7] Trull TJ, Ebner-Priemer UW (2009). Using experience sampling methods/ecological momentary assessment (ESM/EMA) in clinical assessment and clinical research: introduction to the special section. Psychol Assess.

[8] El Dahr Y, Perquier F, Moloney M, Woo G, Dobrin-De Grace R, Carvalho D, Addario N, Cameron EE, Roos LE, Szatmari P, Aitken M (2023). Feasibility of using research electronic data capture (REDCap) to collect daily experiences of parent-child dyads: ecological momentary assessment study. JMIR Form Res.

[9] Baggott C, Gibson F, Coll B, Kletter R, Zeltzer P, Miaskowski C (2012). Initial evaluation of an electronic symptom diary for adolescents with cancer. JMIR Res Protoc.

[10] Knell G, Gabriel KP, Businelle MS, Shuval K, Wetter DW, Kendzor DE (2017). Ecological momentary assessment of physical activity: validation study. J Med Internet Res.

[11] Gaser D, Peters C, Götte M, Oberhoffer-Fritz R, Feuchtinger T, Schmid I, von Luettichau I, Kesting S (2022). Analysis of self-reported activities of daily living, motor performance and physical activity among children and adolescents with cancer: baseline data from a randomised controlled trial assessed shortly after diagnosis of leukaemia or non-Hodgkin lymphoma. Eur J Cancer Care (Engl).

[12] Kampshoff CS, Verdonck-de Leeuw IM, van Oijen MG, Sprangers MA, Buffart LM (2019). Ecological momentary assessments among patients with cancer: a scoping review. Eur J Cancer Care (Engl).

[13] Jarrahi MH, Goray C, Zirker S, Zhang Y, Symon G, Pritchard K, Hine C (2021). Digital diaries as a research method for capturing practices in situ. Research Methods for Digital Work and Organization: Investigating Distributed, Multi-Modal, and Mobile Work.

[14] Sieber C, Chiavi D, Haag C, Kaufmann M, Horn AB, Dressel H, Zecca C, Calabrese P, Pot C, Kamm CP, von Wyl V, Swiss Multiple Sclerosis Registry (2022). Electronic health diary campaigns to complement longitudinal assessments in persons with multiple sclerosis: nested observational study. JMIR Mhealth Uhealth.

[15] de Jager A, Fogarty A, Tewson A, Lenette C, Boydell K (2017). Digital storytelling in research: a systematic review. Qual Rep.

[16] De Vecchi N, Kenny A, Dickson-Swift V, Kidd S (2016). How digital storytelling is used in mental health: a scoping review. Int J Ment Health Nurs.

[17] Rieger KL, West CH, Kenny A, Chooniedass R, Demczuk L, Mitchell KM, Chateau J, Scott SD (2018). Digital storytelling as a method in health research: a systematic review protocol. Syst Rev.

[18] Creswell JW, Plano Clark VL (2018). Designing and Conducting Mixed Methods Research. 3rd edition.

[19] van Manen M (2016). Researching Lived Experience: Human Science for an Action Sensitive Pedagogy.

[20] Doherty K, Balaskas A, Doherty G (2020). The design of ecological momentary assessment technologies. Interact Comput.

[21] Lincoln Y, Guba E (1985). Naturalistic Inquiry.

[22] Bartlett R (2012). Modifying the diary interview method to research the lives of people with dementia. Qual Health Res.

[23] (2020). Interprofessionelle Zusammenarbeit in der Gesundheitsversorgung: erfolgskritische Dimensionen und Fördermassnahmen. Differenzierung, Praxis und Implementierung. Swiss Academy of Medical Sciences.

[24] Hall MA, Dugan E, Zheng B, Mishra AK (2001). Trust in physicians and medical institutions: what is it, can it be measured, and does it matter?. Milbank Q.

[25] Bolger N, Davis A, Rafaeli E (2003). Diary methods: capturing life as it is lived. Annu Rev Psychol.

[26] Seguin M, Mendoza J, Mallari E, Lasco G, Maever L Amit A, Palileo-Villanueva LM, Palafox B, Renedo A, McKee M, Balabanova D (2022). Participant use of digital diaries in qualitative research: a strong structuration analysis. Int J Qual Methods.

[27] Bennett J (2014). Using Diaries and Photo Elicitation in Phenomenological Research: Studying Everyday Practices of Belonging in Place.

[28] Manji K, Hanefeld J, Vearey J, Walls H, de Gruchy T (2021). Using WhatsApp messenger for health systems research: a scoping review of available literature. Health Policy Plan.

[29] Lincoln YS, Guba EG (2004). But is it rigorous? Trustworthiness and authenticity in naturalistic evaluation. New Dir Prog Eval.

[30] Manning K (2016). Authenticity in constructivist inquiry: methodological considerations without prescription. Qual Inq.

[31] Hasal M, Nowaková J, Ahmed Saghair K, Abdulla H, Snášel V, Ogiela L (2021). Chatbots: security, privacy, data protection, and social aspects. Concurr Comput.

[32] Masoni M, Guelfi MR (2020). WhatsApp and other messaging apps in medicine: opportunities and risks. Intern Emerg Med.

[33] Chari A, Gane SB (2018). Instant messaging applications in healthcare: are we harnessing their potential?. BMJ Innov.

[34] Chaix B, Bibault JE, Pienkowski A, Delamon G, Guillemassé A, Nectoux P, Brouard B (2019). When chatbots meet patients: one-year prospective study of conversations between patients with breast cancer and a chatbot. JMIR Cancer.

[35] Singer B, Walsh CM, Gondwe L, Reynolds K, Lawrence E, Kasiya A (2020). WhatsApp as a medium to collect qualitative data among adolescents: lessons learned and considerations for future use. Gates Open Res.

[36] Lindner OC, Phillips B, McCabe MG, Mayes A, Wearden A, Varese F, Talmi D (2014). A meta-analysis of cognitive impairment following adult cancer chemotherapy. Neuropsychology.

[37] Durán-Gómez N, López-Jurado CF, Nadal-Delgado M, Pérez-Civantos D, Guerrero-Martín J, Cáceres MC (2022). Chemotherapy-related cognitive impairment in patients with breast cancer based on functional assessment and NIRS analysis. J Clin Med.

[38] Herron R, Dansereau L, Wrathall M, Funk L, Spencer D (2019). Using a flexible diary method rigorously and sensitively with family carers. Qual Health Res.

[39] Vaghefi I, Tulu B (2019). The continued use of mobile health apps: insights from a longitudinal study. JMIR Mhealth Uhealth.

[40] Druce KL, Dixon WG, McBeth J (2019). Maximizing engagement in mobile health studies: lessons learned and future directions. Rheum Dis Clin North Am.

[41] Weis J, Wolf LR, Boerries M, Kassahn D, Boeker M, Dresch C (2023). Identification of the needs and preferences of patients with cancer for the development of a clinic app: qualitative study. JMIR Cancer.

[42] Alpers GW, Frey L, Tessmer-Petzendorfer S, Klingauf A, Schad S (2020). How shall I write to my patient? Data protection in digital communication. Verhaltenstherapie.

[43] Mierzwa S, Souidi S, Conroy T, Abusyed M, Watarai H, Allen T (2019). On the potential, feasibility, and effectiveness of chat bots in public health research going forward. Online J Public Health Inform.

[44] Daniëls NE, Hochstenbach LM, van Zelst C, van Bokhoven MA, Delespaul PA, Beurskens AJ (2021). Factors that influence the use of electronic diaries in health care: scoping review. JMIR Mhealth Uhealth.

