# Supplementary material for: Using a Mobile Messenger Service as a Digital Diary to Capture Patients’ Experiences Along Their Interorganizational Treatment Path in Gynecologic Oncology: Lessons Learned
Source: JMIR Cancer. 2024 Jul 29;10:e52985. doi: 10.2196/52985 (PMC11319886; doi:10.2196/52985)
Supplement: Multimedia Appendix 1 [file cancer_v10i1e52985_app1.docx]

# **Mulimedia Appendix 1:** **Messenger Evaluation Checklist**

The following list identifies the requirements that a secure messaging app must meet and what organizations (i.e., research teams) must look for to be privacy compliant when handling sensitive health data. The list is inspired by the website “Secure Messaging Apps Comparison” (see <https://www.securemessagingapps.com/?utm_source=pocket_mylist> ).

- **Company jurisdiction:** This matters because many countries have laws that demand that encrypted data be able to be decrypted by the government. Many other countries employ vast surveillance networks or have uncomfortably close relationships with companies when it comes to gaining access to customers’ data.
- **Infrastructure jurisdiction:** See above. In order to operate a truely global service, companies may have infrastructure in different regions of the world in order to, for example, provide lower network latency.
- **Implicated in giving customers’ data to intelligence agencies:** Companies can be forced by law to give customers’ data to intelligence agencies. Other known methods by which these agencies can get customers’ data include coercion, hacking, planting an employee, or simply asking nicely. The term “intelligence agencies” is used to refer to any government agency. “customers’ data” here means customers’ content/messages (data, not metadata).
- **Surveillance capability built into the app?:** This matters because some jurisdictions mandate that certain systems must have surveillance access for governments.
- **Does the company provide a transparency report?:** Many companies periodically publish a transparency report. This details what type of requests have been received from governments, how many requests were made, how many customers were affected, etc.
- **Funding:** This matters because “money talks”, as the saying goes. If the company or person behind the money is likely to have reason not to protect customers’ privacy, it’s important to know. This could be indicative of the company not doing as they say (Google, WhatsApp, for example) or changing their mind once they’ve onboarded enough customers from whom they can make money.
- **Company collects customers’ data:** This matters because many companies use customers’ data for advertising, for improving their services, or simply to sell to other companies.
- **App collects customers’ data:** This matters because many companies use customers’ data for advertising, for improving their services, or simply to sell to other companies.
- **Is encryption turned on by default?:** Self-explanatory.
- **Cryptographic primitives (key derivation, symmetric encryption, authentication and integrity):** Specific key derivation, encryption, and hashing algorithms are considered secure by cryptographers. It’s important that algorithms without known weaknesses are used.
- **Are the app and server completely open source?:** This matters because a fully open source app can be audited by the industry. Open-source code leads to near full transparency. Likewise, we can find any vulnerabilities in the software, weaknesses in the implementation, or design deficiencies. The server code must also be open source; this is because all apps use a central directory service to match users. Vulnerabilities and backdoors could exist in these directory services.
- **Are reproducible builds used to verify apps against source code?**:

Are you sure that the app you downloaded from Google and/or Apple is using the exact code source that the developers published?

- **Can you sign up to the app “anonymously”?**: This matters because many people have good reasons for needing to remain anonymous. Having to provide a unique ID of some kind — a cellphone number, email address, etc. — means giving away something that could be used to track you.
- **Can you manually add contacts without needing to trust a directory server?**: Some apps require that you register yourself with a cellphone number or email address. This data is stored on the company’s servers (with one-way encryption [a hash] hopefully). It matches phone numbers and/or email addresses in your contact list (assuming that you allow the app access to it) so that you know who else uses the same app. However, how do you know that you have been “matched” with the correct person? That the company hasn’t matched you with someone else (e.g., an intelligence agent)? Some apps allow you to manually add a contact without needing to trust that a third party correctly matches you. You can add people anonymously, thereby increasing your privacy.
- **Can you manually verify contacts’ fingerprints?** In order to ensure that you’re talking to whom you believe you are, it’s important that apps support the verification of users’ fingerprints. If you cannot manually verify fingerprints within the app — by scanning a QR code, or by publishing your fingerprint, or by sending your fingerprint via another medium, or simply reading it over the phone — then your messages could be intercepted by what is called a “man in the middle (MITM)” attack.
- **Could the directory service be modified to enable a MITM attack (especially when first adding a contact)?**
- **Do you get notified if a contact’s fingerprint changes?:** A contact’s fingerprint changes when they reinstall the app/their phone without having backed up (if it’s possible) their ID and encryption key. If the ID and encryption key were not backed up, or the entire phone was not reinstalled, then the app will regenerate a new ID and encryption key, which is represented by a fingerprint. Hence a new fingerprint will be generated. However, a contact’s new fingerprint could also be a sign of a man in the middle attack. Hence you should re-verify your contacts if their fingerprint changes.
- **Is any personal information (cellphone number, email address, contact list, etc.) hashed?:** If data is hashed, it’s unreadable to companies. If, for example, a phone number is hashed, it’s given a unique, irreversible representation that is essentially gibberish. Each phone number will always have a unique representation (hash).This method can be used to protect contact lists. Instead of uploading a list of your contacts, it’s more secure to upload a hash of each contact
- **Does the app generate & keep a private key on the device itself?** In order for end-to-end encryption, the encryption key must be generated and kept on the device itself. If a company has access to the encryption key, then it’s not secure.
- **Can messages be read by the company?:** self-explanatory. For apps that can do both unencrypted and encrypted messages (Telegram, Google Allo, etc.), the answer is “Yes”.
- **Does the app enforce perfect forward secrecy? (At the message encryption level, not transport over networks)** Each message that’s sent should be protected by a unique encryption key (often called a session key). This way if the encryption key on the device is compromised, it doesn’t necessarily compromise past messages (that would have been encrypted with a unique encryption key).
- **Does the app encrypt metadata?** Metadata can include the date and time you sent a message, your location, and to whom you sent a message. (Basically any information about the information that you’re sending.) This is important because this data can reveal an awful lot about you. It’s also targeted by law enforcement agencies.
- **Does the app use TLS/Noise to encrypt network traffic?** It’s important that al communication between the app and its servers is encrypted over the Internet. This is the same technology that banks, Google, etc. use.
- **Does the app use certificate pinning?** This ensures that TLS connections only happen between the app and the company’s servers. Specifically, the app only trusts TLS certificates that come from the company (the public keys of those specific certificates are “pinned” in the app).
- **Does the app encrypt data on the device? (iOS and Android only assessed):** Encrypting devices (and the data in memory when devices are locked) is important so that the data on them cannot be read without the correct passcode.
- **Does the app allow a secondary form of authentication?** Some of the apps provide a secondary form of authentication — either a password/code or a fingerprint. This provides an extra level of access control to the data that’s held in the app.
- **Are messages encrypted when backed up to the cloud?** Some apps offer end-to-end encryption that does not encrypt the messages when they are backed up to the cloud. For example, WhatsApp messages are stored in clear text (readable by Facebook) when iCloud is used to back up a device.
- **Does the company log timestamps/IP addresses?** Some companies (WhatsApp, for example) retain date and timestamp information of messages.
- **Have there been a recent code audit and an independent security analysis?** It’s important that each app has been independently tested. Anyone can create a system that they themselves cannot break. This can also help us trust closed sourced apps, such as Threema.
- **Is the design well documented?** It’s important that the clients, Application Programming Interfaces (APIs), servers, directory servers, and messaging algorithms are all designed correctly. Having design documents published enables experts to check that all of these have been designed correctly.
- **Does the app have self-destructing messages?** This means that messages will be automatically deleted after a certain period of time.
