# Supplementary material for: Using a Mobile Messenger Service as a Digital Diary to Capture Patients’ Experiences Along Their Interorganizational Treatment Path in Gynecologic Oncology: Lessons Learned
Source: JMIR Cancer. 2024 Jul 29;10:e52985. doi: 10.2196/52985 (PMC11319886; doi:10.2196/52985)
Supplement: Multimedia Appendix 2 [file cancer_v10i1e52985_app2.docx]

**Multimedia Appendix 2.** Exemplary analysis of 5 messengers.

| Messenger | | Signal (Signal Messenger LLC) | Telegram (Telegram Messenger Inc) | Threema (Threema GmbH) | | Facebook and WhatsApp (Meta platforms, Inc) | Wire (Wire Swiss GmbH) |
| --- | --- | --- | --- | --- | --- | --- | --- |
| **Overview** | | | | | | | |
|  | Is the application recommended to secure messages and attachments? | - Yes | - No | | - Yes | - No | - Yes |
|  | Main reasons why the application is not recommended or improvements recommended for the application | - Remove the mandatory requirement for users to sign up with a mobile number - Provide more comprehensive independent assessments of security or privacy | - Bespoke cryptography - Encryption is not enabled by default - User data (phone numbers and other contact information) is not protected | | - Make APIs^a^ and server code open source - Implement perfect forward secrecy at the end-to-end encryption layer - Provide more comprehensive independent assessments of security/privacy | - Named as NSA^b^ partner in Snowden revelations - Messages can be read by Facebook if marked as *abusive* - Makes money from personal data - Not all data are protected - No independent and recent code audit and security analysis - Closed source | - Further limit metadata storage and logging - Provide more comprehensive independent assessments of security/privacy |
| **Details** | | | | | | | |
|  | Company jurisdiction | - United States | - United States/United Kingdom/Belize/ United Arab Emirates | | - Switzerland | - United States | - United States/ Switzerland |
|  | Infrastructure jurisdiction | - United States | - United Kingdom/Singapore/United States/ Finland | | - Switzerland | - United States (unsure of other locations) | - European Union |
|  | Is the application implicated in giving customers’ data to intelligence agencies? | - No | - No | | - No | - Yes | - No |
|  | Is surveillance capability built into the application? | - No | - No | | - No | - No | - No |
|  | Does the company provide a transparency report? | - Yes | - No | | - Yes | - Yes | - Yes |
|  | Funding | - Freedom of the Press Foundation/the Knight Foundation/the Shuttleworth Foundation/the Open Technology Fund / Signal Foundation (Brian Acton) | - Pavel Durov | | - User pays/Afinum Management AG | - Facebook | - Janus Friis/Iconical/Zeta Holdings Luxembourg/Morpheus Ventures |
|  | Does the company collect customers’ data? | - No | - Yes | | - No | - Yes | - No |
|  | Does the application collect customers’ data? | - Contact information | - Contact information/contacts/identifiers | | - Contact information/identifiers/diagnostics(contact information not sent when using anonymously) | - Purchases/financial information/location/contact information/contacts/user content/identifiers/use data/diagnostics | - Contact information/identifiers/use data/diagnostics |
|  | Are user data or metadata sent to the parent company and/or third parties? | - Minimal(mandatory to send mobile number to a third party for registration and recovery) | - Yes | | - No (optional to send mobile number to a third party for registration) | - Yes | - Yes |
|  | Is encryption turned on by default? | - Yes | - No | | - Yes | - Yes (if the device supports it) | - Yes |
|  | Cryptographic primitives | - Curve25519/AES-256/HMAC-SHA256 | - RSA 2048/AES 256/SHA-256 | | - Curve25519 256/XSalsa20 256/Poly1305-AES 128 | - Curve25519/AES-256/HMAC-SHA256 | - Curve25519/ChaCha20/HMAC-SHA256 |
|  | Are the application and server completely open source? | - Yes | - No (clients and API only) | | - No (applications only) | - No | - Yes |
|  | Are reproducible builds used to verify applications against the source code? | - Android only | - iOS and Android | | - Android only | - No | - No |
|  | Can you sign up to the application anonymously? | - No | - No | | - Yes | - No | - No |
|  | Can you add a contact without needing to trust a directory server? | - No | - No | | - Yes | - No | - No |
|  | Can you manually verify contacts’ fingerprints? | - Yes | - No (session only; does not provide users’ fingerprint information) | | - Yes | - Yes | - Yes |
|  | Can the directory service be modified to enable a MITM^c^ attack? | - Yes | - Yes | | - Yes | - Yes | - Yes |
|  | Do you get notified if a contact’s fingerprint changes? | - Yes | - No (session only; does not provide users’ fingerprint information) | | - Yes | - No (setting turned off by default) | - Yes (if the contact was previously verified) |
|  | Is personal information (eg, mobile number and contact list) hashed? | - Mostly | - No | | - Yes | - No | - Mostly |
|  | Does the application generate and keep a private key on the device itself? | - Yes | - Yes | | - Yes | - Yes | - Yes |
|  | Can messages be read by the company? | - No | - Yes | | - No | - Yes | - No |
|  | Does the application enforce perfect forward secrecy? | - Yes | - No (session keys do change after being used 100 times) | | - No | - Yes | - Yes |
|  | Are metadata encrypted? | - Yes | - No | | - Yes | - No | - Mostly |
|  | Does the application use TLS^d^/noise to encrypt network traffic? | - Yes | - No | | - Yes | - Yes | - Yes |
|  | Does the application use certificate pinning? | - Yes | - Unknown | | - Yes | - Yes (using the Signal Protocol, formerly TextSecure Protocol) | - Yes |
|  | Does the application encrypt data on the device? (iOS and Android only) | - Yes (if passphrase is enabled) | - No (regular chats are not end-to-end encrypted; only secret ones are; Telegram group chats cannot be end-to-end encrypted) | | - iOS: yes (if passphrase is enabled) - Android: yes (if master key is set in the application) | - Yes (using the Signal Protocol, formerly TextSecure Protocol) | - Yes |
|  | Does the application allow a secondary factor of authentication? | - Yes | - Yes | | - Yes | - Yes | - Yes |
|  | Are messages encrypted when backed up to the cloud? | - N/A^e^; Signal is excluded from iCloud/iTunes and Android backups | - Unknown (messages from secret chats are not stored after delivery) | | - Yes | - iOS: yes - Android: yes | - N/A; Wire is excluded from iCloud/iTunes and Android backups |
|  | Does the company log time stamps/IP addresses? | - No | - Yes | | - No | - Yes | - Some |
|  | Have there been a recent code audit and an independent security analysis? | - Yes (October 2014) | - Yes (November 2015) | | - Yes (October 2020) | - No | - Yes (March 2018) |
|  | Is the design well documented? | - Somewhat: whitepapers or source code available and usable for experts | - Somewhat: whitepapers or source code available and usable for experts | | - Somewhat: whitepapers or source code available and usable for experts | - Somewhat: whitepapers or source code available and usable for experts | - Somewhat: whitepapers or source code available and usable for experts |
|  | Does the application have self-destructing messages? | - Yes | - Yes | | - No | - Yes | - Yes |

^a^API: application programming interface.

^b^NSA: National Security Agency.

^c^MITM: man-in-the-middle.

^d^TLS: transport layer security.

^e^N/A: not applicable.
